# A Microfluidic Dialysis Chip for Continuous Purification of Lipid Nanoparticles

**DOI:** 10.1002/smtd.70976

**Published:** 2026-08-18

**Authors:** Da Zou, Zhenwei Lan, Jingwen Liu, Sha Liu, Song Jin, Yang Li, Phillip Elliott, Jan Bekker, Chun‐Xia Zhao

**Affiliations:** ^1^ School of Chemical Engineering College of Engineering and Information Technology Adelaide University Adelaide South Australia Australia; ^2^ Biocina Thebarton South Australia Australia

**Keywords:** dialysis, lipid nanoparticles, mass transfer, microfluidic, mRNA delivery

## Abstract

Synthesis of lipid nanoparticles (LNPs) for mRNA delivery is now standardized and automated. In contrast, post‐synthesis purification—ethanol removal, clearance of unencapsulated components, and buffer exchange—remains less controlled. Conventional bulk dialysis is time‐consuming, whereas centrifugal ultrafiltration, although rapid, can result in reduced particle recovery and mRNA preservation; both of these laboratory‐scale approaches present limitations for reproducible high‐throughput processing. Here we report a 3D‐printed microfluidic dialysis chip for continuous LNP purification. Mirrored serpentine sample and wash‐buffer channels run counter‐current across a clamped, interchangeable membrane in a three‐layer printed chip. A mass‐transfer model relating ethanol removal to the number of transfer units (NTU) identified channel height as the principal geometric design parameter under the fixed‐flow‐rate conditions tested. At a 100 kDa cut‐off and 25 µL/min, the chip removed >99% ethanol and adjusted pH from 4.0 to 7.4, processing 1 mL in 40 min at steady state. Membrane choice set a trade‐off: Polyethersulfone removed ethanol faster, whereas regenerated cellulose gave the highest particle recovery (83%). Förster resonance energy transfer (FRET) confirmed preserved LNP integrity, and on‐chip purified mRNA‐LNPs matched bulk dialysis in transfection efficiency. Serial or parallel configurations offer a gentle, scalable route to LNP purification.

## Graphical abstract

A 3D‐printed microfluidic dialysis chip enables gentle, continuous‐flow purification of lipid nanoparticles (LNPs). A number‐of‐transfer‐units (NTU)‐based mass‐transfer model guides device design and identifies channel height as the principal geometric parameter controlling ethanol removal and buffer exchange. Under optimized conditions, the chip removes >99% ethanol, achieves effective buffer exchange, and preserves LNP structural integrity and transfection efficiency, providing a potentially scalable platform for LNP purification.

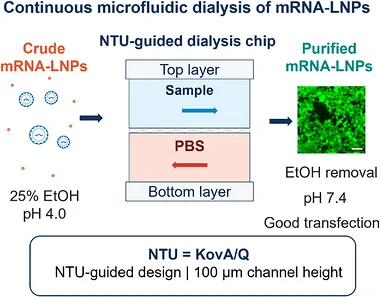

## 1 Introduction

Nucleic acid‐based therapies have attracted significant interest in the past years. However, their clinical translation has been limited until lipid nanoparticles (LNPs) emerged as an effective delivery platform that protects nucleic acids from enzymatic degradation and promotes cellular uptake [1, 2]. The clinical success of mRNA vaccines against COVID‐19 has demonstrated the importance of scalable and reproducible LNP manufacturing processes [3].

Unlike small‐molecule drugs or antibodies, LNPs are colloidal systems whose biological performance depends on multiple physicochemical attributes, including particle size, size distribution, and encapsulation efficiency. As a result, purification is particularly challenging. In addition to removing residual ethanol and unencapsulated lipids or mRNAs, while enabling buffer exchange, purification processes must preserve these critical quality attributes. Residual impurities can induce cytotoxicity, trigger immune responses, and compromise storage stability [4]. Efficient purification that simultaneously removes impurities and maintains particle integrity is thus essential for ensuring product safety, stability, and batch‐to‐batch consistency.

Traditional purification methods include bulk dialysis and ultrafiltration [5]. Bulk dialysis, while simple and widely available, is time‐consuming and difficult to scale. Ultrafiltration is practiced in configurations that differ in scalability: tangential‐flow filtration (TFF) is readily scalable to high‐throughput processing [6], whereas centrifugal ultrafiltration, the laboratory‐scale format evaluated in this study, is a batch process. In the present comparison, centrifugal ultrafiltration was rapid but resulted in lower particle recovery and mRNA preservation (Section 3.3). Statements regarding limited scalability and recovery in this work therefore refer specifically to centrifugal ultrafiltration. These challenges have prompted the development of alternative methods for applications requiring rapid and gentle purification [7].

Microfluidic platforms offer a promising alternative for nanoparticle purification due to their laminar flow regime, short diffusion distances, and precise control over flow dynamics [8, 9]. These features accelerate mass transfer while minimizing shear stress, making microfluidic systems well‐suited for handling shear‐sensitive particles such as LNPs [10, 11]. Previous work has demonstrated microfluidic buffer exchange for various nanoparticle systems [12, 13], but systematic design optimization guided by mass transfer principles remains underexplored.

In this work, we develop a microfluidic dialysis chip for LNP purification and establish a mass transfer framework to guide its design. The study makes four specific contributions: (i) a three‐layer chip architecture with serpentine counter‐current flow that maximizes membrane contact area; (ii) a number‐of‐transfer‐units (NTU)‐based model linking channel geometry and operating conditions to purification efficiency; (iii) systematic optimization identifying channel height, characterized by the ratio of residence time to transverse‐diffusion time (τ_res_/τ_d_), as the principal geometric design parameter under the fixed‐flow‐rate conditions tested; and (iv) a comparative evaluation against bulk dialysis and centrifugal ultrafiltration, demonstrating that microfluidic dialysis achieves higher particle recovery at a comparable processing speed.

## 2 Materials and Methods

### 2.1 Materials

Regenerated cellulose (RC) membranes with molecular weight cut‐offs (MWCO) of 10 and 100 kDa were purchased from Merck Millipore (Ultracel flat‐sheet regenerated‐cellulose discs, cut to fit the chip: PLGC15005 for the 10 kDa and PLHK15005 for the 100 kDa membrane). Polyethersulfone (PES) dialysis membrane with 10 and 100 kDa was obtained from Amicon. The nominal membrane thicknesses were approximately 130 µm for PLGC 10 kDa RC, 230 µm for PLHK 100 kDa RC, and 280 µm for PES. 1,2‐distearoyl‐sn‐glycero‐3‐phosphocholine (DSPC), cholesterol, heptadecan‐9‐yl 8‐((2‐hydroxyethyl)(6‐oxohexyl)amino)octanoate (SM‐102), and 1,2‐dimyristoyl‐sn‐glycerol‐[methoxy(polyethylene glycol)‐2000] (PEG‐DMG‐2000) were purchased from SINOPEG BioTech (Xiamen Co., Ltd., Xiamen, Fujian, China). All other general reagents were supplied by Sigma‐Aldrich.

### 2.2 Preparation of LNPs

LNPs were prepared using a 3D‐printed microfluidic mixer. The mixer consists of two inlet channels that merge at a Y‐junction into a downstream serpentine mixing section followed by a single outlet. Based on the computer‐aided design (CAD) model, all channels, including the two inlet branches, the post‐junction channel, and the serpentine mixing section, have a uniform width of 300 µm and a depth of 100 µm. The inlet branch length is 3 mm, and the serpentine mixing section has an overall length of 9.5 mm. The serpentine geometry is introduced to extend the mixing path after the Y‐junction within a compact device footprint. The lipid components (SM‐102, DSPC, cholesterol, and PEG‐DMG‐2000 at a molar ratio of 50:10:38.5:1.5) were dissolved in ethanol, and mRNA was diluted in citrate buffer (10 mM, pH 4.0). The two phases were introduced into the mixer at a volumetric flow‐rate ratio of 1:3 (organic: aqueous) at a total flow rate of 1 mL/min, with a nitrogen‐to‐phosphate (N/P) ratio of 6. After synthesis, the crude LNP suspension containing approximately 25% (v/v) residual ethanol was collected for subsequent purification. The size and polydispersity index (PDI) of LNPs were measured using a dynamic light scattering (DLS) method (Malvern Instruments, Worcestershire, UK). Transmission electron microscopy (TEM) was applied to observe the morphology of the LNPs. Encapsulation efficiency of mRNA‐loaded LNP was measured using a RiboGreen assay (Thermo Fisher Scientific). To determine the total mRNA amount, 1% Triton X‐100 was added to dissolve LNPs and release the encapsulated RNA, followed by RiboGreen staining and fluorescence measurement using a plate reader.

### 2.3 Fabrication of Dialysis Device

The dialysis device was fabricated using a CADworks3D ProFluidics 285D digital light processing (DLP) 3D printer. The top and bottom chip modules were printed separately using a UV‐curable resin, followed by isopropanol rinsing to ensure the channel was smooth. Each printed module contains a raised or recessed frame around the channel region, allowing the two layers to interlock during assembly. This frame‐to‐frame fitting helps maintain alignment between the upper and lower channels and improves sealing around the membrane. Before assembly, the dialysis membrane was pre‐wetted with phosphate‐buffered saline (PBS) and cut to match the frame dimensions. The membrane was then carefully placed onto the channel region using tweezers, ensuring complete coverage of the channel area. After membrane placement, the top and bottom chip modules were assembled together and secured using ten screws and nuts. This mechanical fixation provided stable sealing and enabled leak‐free operation during dialysis.

### 2.4 In Vitro Study

To test the cellular uptake and potential cytotoxicity of the LNPs, HEK293 cells were used as an in vitro model. Cells were incubated in Dulbecco's Modified Eagle Medium (DMEM) supplemented with 10% fetal bovine serum (FBS) as well as 100 U/mL penicillin and 100 µg/mL streptomycin. Cultures were incubated at 37 °C in a humidified atmosphere with 5% CO_2_.

For cell transfection tests, HEK293 cells were seeded on sterilized 24‐well plates at a density of 1 × 10^5^ cells per well. After overnight attachment, cells were treated with different mRNA‐loaded LNPs diluted in PBS. Following 24 h of incubation, cells were washed three times with PBS before imaging. Imaging was performed using a fluorescence microscope with excitation at 488 nm for enhanced green fluorescent protein (eGFP) and 550 nm for mCherry.

Flow cytometry analysis was performed by seeding HEK293 cells into 24‐well plates (2 × 10^5^ cells/well) and incubating overnight. Cells were then treated with mRNA‐loaded LNPs at the same concentration and incubated for 24 h. Post‐treatment, cells were washed with PBS, detached using trypsin, and resuspended in PBS for analysis using a flow cytometer (Beckman Coulter, USA). Fluorescence signals were recorded using the PE channel for mCherry‐expressing cells and the FITC channel for eGFP‐expressing cells. Unless otherwise stated, the LNPs used for the cell‐based studies were purified on‐chip using a polyethersulfone membrane (100 kDa MWCO) at a sample flow rate of 25 µL/min, and bulk‐dialysis‐purified LNPs were prepared in parallel for comparison. Cells were treated with 50 ng mRNA per well. For luciferase‐loaded LNPs, reporter expression was quantified using a substrate‐based luciferase assay: after 24 h incubation, luciferase substrate was added, and luminescence was measured using a microplate reader.

Cell viability assay. Cytotoxicity of the encapsulated LNP formulations was assessed using a water‐soluble tetrazolium salt (WST‐8) assay. HEK293 cells were seeded into 96‐well plates at a density of 1 × 10^4^ cells per well and allowed to attach for 24 h. Then, the medium was replaced with LNP‐containing samples (prepared via different purification methods), and cells were incubated for another 24 h. After treatment, 20 µL of WST‐8 reagent was added to each well and incubated for 4 h. Absorbance at 490 nm was measured using a microplate reader to evaluate cell viability.

### 2.5 Ethanol Removal and pH Adjustment Assay

Ethanol concentration in LNP samples before and after dialysis was quantified using a commercial ethanol assay kit (Ethanol Assay Kit, Sigma‐Aldrich), following the manufacturer's instructions. To ensure measurement accuracy, samples with ethanol concentrations above the kit's linear detection range (up to 2% v/v) were appropriately diluted with PBS prior to analysis. Each sample was measured in triplicate. Unless otherwise stated, quantitative results are reported as the mean ± standard deviation of three independent experiments performed using the same device with freshly mounted membranes. This experimental design captures run‐to‐run variability associated with membrane replacement and device reassembly, demonstrating the reproducibility of the clamped membrane configuration. The inlet ethanol concentration was 25% (v/v), and the outlet concentration was measured to determine ethanol‐removal efficiency. For buffer‐exchange evaluation, the pH of LNP samples was measured immediately after dialysis using a calibrated micro pH meter. The change in pH was used as an operational readout of citrate‐to‐phosphate buffer replacement. For comparison with conventional purification, the same crude LNP suspension was also processed by bulk dialysis and by centrifugal ultrafiltration. For bulk dialysis, the sample was loaded into SnakeSkin regenerated‐cellulose dialysis tubing (10 kDa MWCO, Thermo Fisher Scientific) and dialyzed against 2 L of PBS overnight (about 20 h) at 4°C under gentle stirring at 200 rpm. Centrifugal ultrafiltration was performed using a 100 kDa RC Amicon Ultra centrifugal filter. The membrane was pre‐rinsed with 2 mL PBS (1500 × g, 15 min), after which the crude LNP suspension was loaded and concentrated (1200 × g, 10 min). The retentate was then diluted with 1 mL PBS and reconcentrated (800 × g, 10 min) to perform a single buffer‐wash step. This centrifugal ultrafiltration procedure was used as a practical buffer‐exchange comparison and was not intended to represent a pressure‐controlled TFF diafiltration process; therefore, permeate flux and diavolume were not defined. Permeate flux was not directly measured because the device was operated under centrifugal force; therefore, the centrifugal force, processing time, membrane MWCO, and wash volume are reported instead.

### 2.6 Mass Transfer Analysis

To establish a theoretical understanding and optimization of the dialysis process, a one‐dimensional mass transfer model for ethanol removal was developed. The model was subsequently extended to describe buffer exchange behavior in this system.

The system consists of counter‐current flow of the samples and the washing buffer, separated by a dialysis membrane. In this case, we made the following assumptions: (i) steady‐state operation; (ii) negligible axial diffusion relative to convection; (iii) a plug‐flow approximation for bulk concentration evolution along the channel. The wash buffer (PBS) was supplied counter‐currently at a dialysate‐to‐sample flow‐rate ratio of 5:1 (e.g., 125 µL/min against 25 µL/min sample flow). A steady‐state mass balance and convection–diffusion simulation (Figure S4) were used to assess the validity of assuming negligible dialysate ethanol concentration. At the experimentally applied 5:1 flow‐rate ratio, the dialysate ethanol concentration reaches approximately 20% of the sample inlet concentration only at the highest‐concentration region of the channel and remains substantially lower elsewhere. In comparison, a 1:1 flow‐rate ratio results in a much higher dialysate concentration (approximately 82% of the sample inlet value), indicating that the zero‐dialysate assumption would not be appropriate under such conditions. Therefore, the zero‐dialysate approximation is considered reasonable for the selected operating conditions, with deviations reflected in the experimentally determined apparent K_ov_ (Figure S4). Under these assumptions, the differential mass balance for ethanol in the sample channel is written in terms of the local transmembrane concentration difference:

(1) $$QdC=-K_{\mathrm{ov}}\left(C-C_{b}\right)dA$$

where Q is the volumetric flow rate of the sample stream (m^3^/s); C and C_b_ are the local ethanol concentrations in the sample (retentate) and dialysate channels at the same axial position (mol/m^3^), respectively; K_ov_ is the overall mass‐transfer coefficient (m/s); and A is the membrane contact area (m^2^). The dialysate‐side concentration is governed by a companion mass balance. At the applied dialysate‐to‐sample flow‐rate ratio of 5:1, C_b_ remains comparatively low, and Equation (1) may be approximated as *Q* d*C* = −*K*_ov_ *C* d*A* for estimation of an apparent overall coefficient. Integration along the channel length then gives:

(2) $$C_{\mathrm{out}}/C_{\mathrm{in}}=\exp\left(-K_{\mathrm{ov}}A/Q\right)=\exp\left(-\mathrm{NTU}\right)$$

where NTU (number of transfer units) represents the dimensionless mass transfer capacity of the system.

The simplified form is an approximation and is valid only where the local dialysate concentration is small relative to the local sample concentration. Any deviation from this condition is absorbed into the experimentally determined apparent K_ov_. In the strict limit C_b_ → 0, the driving force reduces to C alone and the mass balance becomes independent of the relative flow direction. The difference between co‐current and counter‐current operation therefore arises when the finite dialysate‐side concentration is retained, as in the coupled two‐stream simulations shown in Figures S2 and S4.

The overall mass transfer coefficient (K_ov_) encompasses three resistances in series:

(3) $$1/K_{\mathrm{ov}}=1/k_{s}+1/k_{m}+1/k_{b}$$

where *k*_s_, *k*_m_, and *k*_b_ represent the sample‐side, membrane, and buffer‐side mass transfer coefficients (m/s), respectively. The channel‐side coefficient can be related to the Sherwood number: Sh = *k*_s_ *d*_h_/*D*, where *D* ≈ 1.2 × 10^−^^9^ m^2^/s for ethanol in water at 25°C.

In the present device, the overall coefficient combines three resistances in series (Equation 3), whose relative magnitudes cannot be assumed a priori. Depending on membrane type and MWCO, the dialysis membranes used in this study are approximately 130–280 µm thick, comparable to or greater than the 100 µm channel height. Solute transport across the membrane occurs primarily through diffusion along a tortuous porous pathway, with negligible convective contribution. Therefore, membrane thickness contributes to the overall mass‐transfer resistance and influences the effective mass‐transfer coefficient, with thicker membranes expected to reduce solvent and buffer‐exchange rates while potentially improving nanoparticle retention. The membrane resistance is therefore expected to be comparable to, and possibly larger than, the channel‐side resistance, so no single term dominates. The one inference supported by the experimental data, without deconvoluting the individual resistances, is that because the membrane resistance is essentially independent of channel height, the strong dependence of ethanol removal on H (Section 3.2.1) must originate from the channel‐side transport and residence‐time contributions. Channel height is therefore the principal design variable available for tuning K_ov_, even though it may not represent the largest individual resistance. Because the relative contributions cannot be fully separated without independent characterization, the experimental K_ov_ values are reported as lumped overall coefficients.

A transverse Péclet number was defined to compare axial convection with diffusion across the channel height:

(4) $$Pe_{t}=uH/D$$

At a fixed volumetric flow rate and channel width, u = Q/(WH), so that Pe_t_ = Q/(WD) is independent of channel height (Pe_t_ ≈ 3.5 × 10^2^ for the geometries used here); the influence of channel height is captured instead by the ratio of residence time to transverse‐diffusion time introduced below. The characteristic diffusion time can be estimated as τ_d_ = H^2^/D, which is on the order of several seconds for H = 100 µm. The competition between transverse diffusion and axial convection is captured compactly by the ratio of residence time to transverse‐diffusion time, τ_res_/τ_d_ = LWD/(QH), which decreases as the channel height increases and therefore predicts poorer transverse equilibration, and hence lower ethanol removal, at larger H.

The apparent overall mass transfer coefficient was determined experimentally by rearranging the integrated mass balance:

(5) $$K_{\mathrm{ov}}=-\left(Q/A\right)\ln\left(C_{\mathrm{out}}/C_{\mathrm{in}}\right)$$

The membrane contact area (A = 3.84 cm^2^) was determined from the channel geometry: 24 straight segments of 10 mm × 1 mm and 23 semicircular connectors of 4 mm outer diameter, yielding a total channel length of 38.4 cm.

The mass transfer analysis provides several design guidelines:

1. For >99% ethanol removal (NTU > 4.6), operating conditions should satisfy *K*_ov_ *A*/*Q* > 4.6.
2. Channel height should be minimized to reduce diffusion distance and enhance boundary layer mass transfer.
3. Moderate flow rates (higher τ_res_/τ_d_) favor complete removal; throughput can be increased by parallelization rather than increasing single‐channel flow rate.
4. Membrane selection should balance permeability for small molecules (ethanol) with retention of LNPs.

The mass transfer framework developed above for ethanol can be conceptually extended to buffer exchange, although the underlying transport mechanisms differ. While ethanol removal is governed primarily by molecular diffusion across the membrane, buffer exchange involves the transport of multiple ionic species (e.g., citrate, phosphate, protons) with potentially different diffusion coefficients and membrane permeabilities.

To characterize buffer exchange efficiency, a dimensionless exchange parameter was defined as:

(6) $$\eta =\left(pH_{\mathrm{out}}-pH_{\mathrm{in}}\right)/\left(pH_{\mathrm{target}}-pH_{\mathrm{in}}\right)$$

where pH_in_ is the inlet sample pH (citrate buffer, pH 4), pH_out_ is the measured outlet pH, and pH_target_ is the wash buffer pH (PBS, pH 7.4). Under this operational definition, η = 1 indicates complete pH adjustment to the PBS pH and effective buffer exchange as judged by the outlet pH.

Because pH is logarithmic in hydrogen‐ion activity, η is linear in pH but not in hydrogen‐ion concentration or buffer composition. An outlet pH 0.1 unit below the target corresponds to a hydrogen‐ion activity approximately 26% higher. Accordingly, η is used here only as an operational indicator of pH adjustment during buffer exchange and not as a direct measure of residual citrate or complete buffer replacement.

## 3 Results and Discussion

### 3.1 Design and Fabrication of the 3D‐Printed Microfluidic Dialysis Chip

After microfluidic synthesis, the crude LNP suspension contains approximately 25% (v/v) residual ethanol in acidic citrate buffer (pH 4.0). Both components must be removed before biological applications. Ethanol is cytotoxic and destabilizes LNP structure during storage, while the acidic pH is incompatible with physiological conditions. The objective of the on‐chip dialysis step is therefore: (i) to achieve near‐complete ethanol removal (>99%) and (ii) to exchange the citrate buffer for PBS (pH 7.4), while preserving LNP size, structural integrity, and mRNA encapsulation. To meet these requirements, a microfluidic dialysis chip was designed. We describe below the design rationale, fabrication and assembly process, and performance of the device.

**FIGURE 1:**
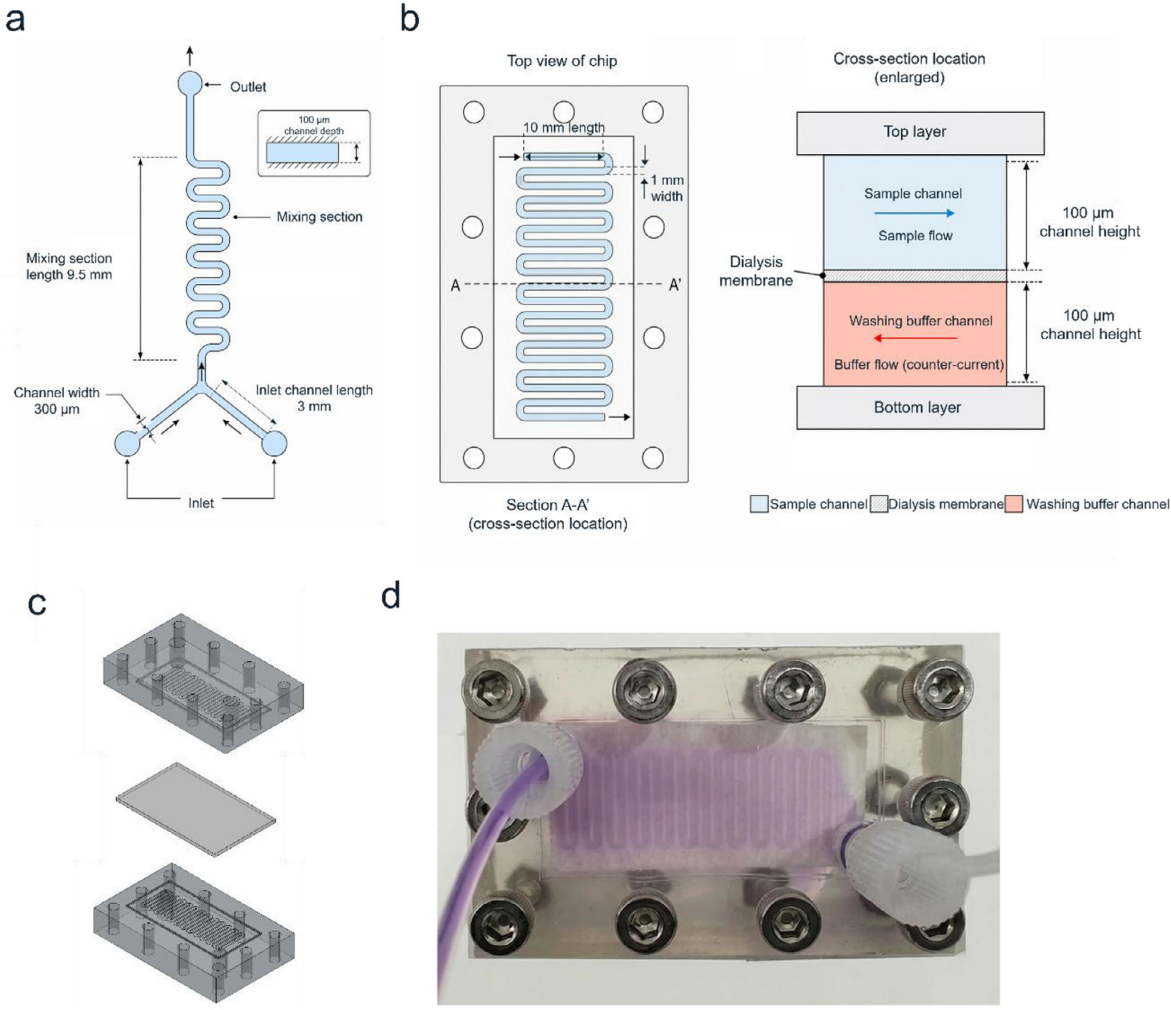
Design and assembly of the microfluidic chip for LNP synthesis and purification. (a) Schematic of the microfluidic synthesis chip. (b) Top‐view layout of the dialysis chip and the corresponding enlarged cross‐sectional schematic. (c) Exploded‐view schematic of the 3D‐printed dialysis module, showing the top layer, the dialysis membrane, and the bottom layer before assembly. (d) Photograph of the assembled dialysis chip connected to tubing during operation.

In the synthesis stage, the lipid‐ethanol phase and the aqueous mRNA‐citrate buffer phase are mixed in a 3D‐printed microfluidic mixer (Figure 1a) as described in Section 2.2. The collected crude LNP suspension is then loaded into the dialysis chip for purification. The dialysis chip adopts a three‐layer sandwich structure (Figure 1b), comprising a sample channel in the top layer and a PBS channel in the bottom layer, separated by a commercially available dialysis membrane clamped between the two channels. Both channels follow an identical serpentine path in a counter‐current configuration. The serpentine design consists of 24 straight segments (each 10 mm long × 1 mm wide) connected by 23 semicircular turns (4 mm outer diameter), yielding a total channel length of 38.4 cm and a membrane contact area of 3.84 cm^2^. The channel height was set at 100 µm (optimized in Section 3.2.1). This geometry was chosen for two reasons. Firstly, the long serpentine path maximizes the membrane contact area and hence the NTU available for ethanol removal. Secondly, the shallow channel height keeps the characteristic diffusion time well below the channel residence time, ensuring efficient solute transport to the membrane surface. The counter‐current configuration was selected because it maintains a favorable concentration gradient across the membrane along the entire channel length, thereby sustaining the mass‐transfer driving force more effectively than co‐current operation. In contrast, the concentration difference decreases progressively in co‐current flow as the two streams move in the same direction and approach equilibrium. A convection–diffusion simulation of the two‐channel contactor further confirms the advantage of this configuration. Under otherwise identical operating conditions, the simulated outlet ethanol concentration was approximately 2% for counter‐current operation, compared with approximately 17% for co‐current operation (Figure S2), demonstrating substantially enhanced solvent removal efficiency. Both the sample and wash‐buffer channels are serpentine channels with identical geometries, mirrored across the membrane. Therefore, the wash buffer does not flow through a separate straight channel; instead, it follows the same serpentine path in the opposite direction to the sample stream, establishing a counter‐current configuration along the entire membrane interface. The corresponding internal channel volume is approximately 38 µL, giving a residence time of ∼90 s at 25 µL/min. The processing time reported below therefore represents steady‐state throughput. Channel filling, equilibration, and flushing contribute only a small fixed overhead (on the order of one residence time), and any sample loss to the channel dead volume is limited to the system hold‐up volume (≈38 µL, i.e., below 2% of a 2 mL batch).

Initial prototypes fabricated using soft lithography with PDMS suffered from leakage above 10 µL/min due to inadequate membrane sealing. Switching to DLP 3D printing with a rigid UV‐cured resin resolved this issue. The top and bottom channel layers were printed separately and assembled with the dialysis membrane sandwiched between them, as detailed in Section 2.3. Mechanical clamping with stainless‐steel fasteners provided leak‐free operation at flow rates up to 500 µL/min (Figure 1c,d). Channel heights below 100 µm were not pursued because they fall outside the reliable fabrication window of our DLP printing and clamped‐membrane assembly approach. At smaller channel heights, maintaining a consistent leak‐free seal across the 38.4 cm serpentine footprint becomes increasingly challenging, while the increased hydraulic resistance and potential for membrane deflection into the channel may compromise flow stability. These limitations represent practical fabrication and operation constraints rather than a limitation of the underlying design principle. Furthermore, the model indicates diminishing returns below 100 µm, as the transverse diffusion time is already substantially shorter than the residence time at this channel height. The transparent resin also permits optical access for flow visualization, while standard tubing connections enable straightforward integration with syringe pumps.

### 3.2 Parameter Optimization and Design Guidelines

The mass transfer model developed in Section 2.6 predicts that purification efficiency is governed by the NTU, defined as:

(7) $$\mathrm{NTU}=K_{\mathrm{ov}}A/Q$$

where K_ov_ is the overall mass transfer coefficient (m/s), accounting for the combined resistances of the sample‐side boundary layer, the membrane, and the buffer‐side boundary layer (Equation 3). A is the membrane contact area, and Q is the volumetric flow rate of the sample stream (m^3^/s). A higher NTU corresponds to greater ethanol removal. NTU > 4.6 is required for >99% removal. Since K_ov_ is influenced by channel geometry (through the boundary‐layer thickness and hence the Sherwood number) while A and Q are set by channel dimensions and flow rate, systematic variation of these parameters allows identification of the dominant design variables.

#### 3.2.1 Channel Height

**FIGURE 2:**
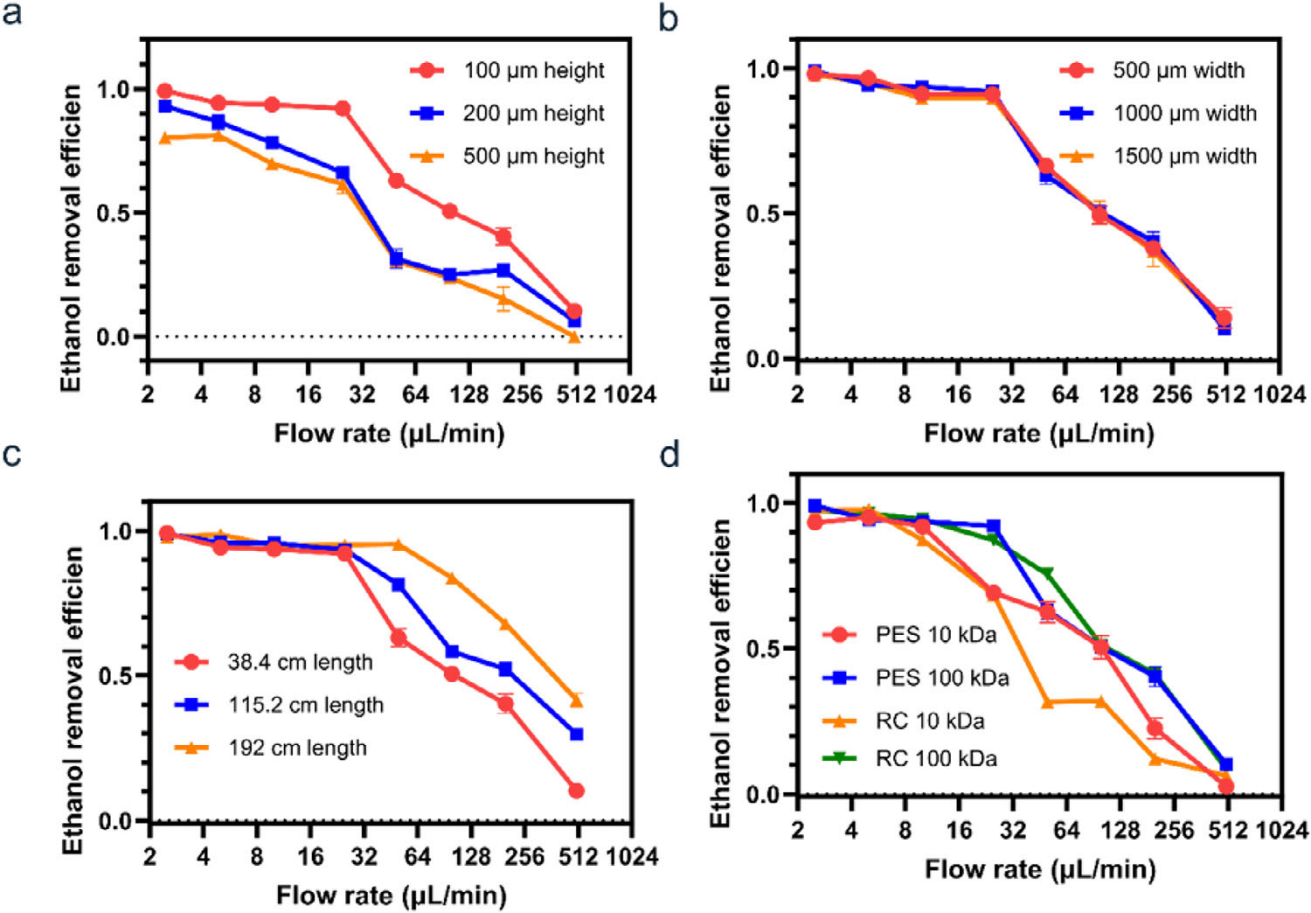
Effects of structural parameters and membrane properties on ethanol removal performance. (a) The relationship between ethanol removal efficiency and channel height. (b) The relationship between purification performance and channel width. (c) The relationship between purification performance and channel length. (d) The relationship between ethanol removal efficiency and dialysis membrane material. Unless otherwise stated, measurements were made at a sample flow rate of 25 µL/min with a dialysate‐to‐sample flow‐rate ratio of 5:1; data are mean ± standard deviation of three independent experiments.

Three chips with heights of 100, 200, and 500 µm were tested at fixed width (1 mm) and length (38.4 cm) with a PES membrane (100 kDa MWCO), the three heights being compared at matched volumetric flow rate rather than at matched residence time (Figure 2a). Ethanol removal efficiency decreased markedly with increasing height. Because the three heights were compared at matched volumetric flow rate, the residence time τ_res_ = LWH/Q increases only linearly with height, whereas the transverse diffusion time τ_d_ = H^2^/D increases quadratically. Their ratio, τ_res_/τ_d_ = LWD/(QH), therefore decreases from approximately 11 at H = 100 µm (τ_d_ ≈ 8 s and residence time approximately 92 s) to 5.5 at 200 µm and 2.2 at 500 µm, indicating progressively less complete transverse equilibration (Figure S3).

At fixed volumetric flow rate, increasing channel height also reduces the near‐membrane wall shear rate, ${\dot{\gamma }}_{w}$ = 6Q/(WH^2^), from approximately 250 s^−1^ at H = 100 µm to 62.5 s^−1^ at 200 µm and 10 s^−1^ at 500 µm. This reduction is expected to decrease the channel‐side boundary‐layer mass‐transfer coefficient, consistent with Lévêque/Graetz‐type transport scaling. The observed height dependence therefore reflects combined changes in transverse diffusion and near‐membrane hydrodynamics. Because matched‐shear‐rate experiments were not performed, these contributions cannot be resolved independently from the present data. Under the fixed‐flow‐rate conditions tested, both effects favor the smallest reliably fabricable channel height. As pH adjustment relies on the transverse transport of buffer species and protons across the membrane, increasing the channel height is expected to reduce pH‐adjustment efficiency, similar to its effect on ethanol removal, due to the increased diffusion distance and longer mass‐transfer time. The shallow 100 µm channel that meets the ethanol‐removal criterion is therefore also most favorable for achieving effective buffer exchange (η → 1).

#### 3.2.2 Channel Width

Chips with widths of 500, 1000, and 1500 µm showed no significant difference in removal efficiency (Figure 2b). This result is consistent with the model: mass transfer is limited by diffusion across the channel height, not the width. Lateral concentration gradients equilibrate rapidly because width does not affect the rate‐limiting vertical transport step. Width can be selected based on throughput and fabrication considerations, and it does not significantly affect mass transfer performance.

#### 3.2.3 Channel Length

Extending length from 38.4 to 115.2 cm improved removal efficiency at fixed flow rate (Figure 2c). Longer channels increase membrane area A and hence NTU = K_ov_A/Q. For applications requiring NTU > 4.6 (>99% removal) at higher flow rates, modules can be connected in series rather than fabricating impractically long single channels. Increasing channel length or the number of serial modules is a good approach to achieve target NTU at the desired flow rate. From a mass‐transfer perspective, serially connected modules are approximately equivalent to a single channel with the same total length, because both increase the available membrane area (A) and consequently the NTU. However, they are not fully equivalent in practice. Each inter‐module connection introduces additional dead volume and pressure drop, may transiently disturb the developing concentration boundary layer, and requires consistent membrane alignment and sealing to maintain reproducible performance. Serial concatenation is nonetheless preferable to a single impractically long channel, because it preserves reliable membrane sealing and allows individual modules to be validated, at the cost of modest additional hold‐up volume.

#### 3.2.4 Membrane Material and MWCO

Two membrane materials were evaluated, PES and RC, each with two MWCO values (10 and 100 kDa). PES is widely used for its high hydraulic permeability and chemical resistance, while RC is favored for its low nonspecific adsorption, which is beneficial for shear‐sensitive biologics. The comparison aimed to identify which combination best balances ethanol removal speed against LNP particle recovery. Under identical conditions, PES membranes achieved higher ethanol removal than RC membranes (Figure 2d), consistent with a higher effective K_ov_ in the present device. Because ethanol (46 Da) is far below the 100 kDa cut‐off, removal is governed by the effective diffusive pathway through the membrane rather than by sieving. The faster ethanol removal observed for PES, despite its greater nominal thickness (∼280 µm compared with ∼230 µm for PLHK RC), likely arises from differences in effective diffusivity, including membrane porosity, tortuosity, swelling behavior, and microstructural characteristics, rather than MWCO alone. Because ethanol (46 Da) is substantially smaller than the membrane MWCO (∼100 kDa), its transport is not expected to be limited by molecular sieving. This interpretation applies specifically to the membranes evaluated in this study and does not imply that PES membranes are generally more permeable than RC membranes. The lower particle loss observed with RC (Section 3.3) is consistent with its lower nonspecific adsorption behavior compared with PES [14]. Increasing MWCO from 10 to 100 kDa further improved removal kinetics for both materials, consistent with reduced membrane resistance (k_m_). However, PES exhibited slightly higher particle loss than RC (quantified in Section 3.3), indicating a trade‐off between removal speed and particle recovery. In practice, PES (100 kDa) is recommended when rapid ethanol removal is the priority, while RC (100 kDa) is preferable when minimizing particle loss is the primary concern.

#### 3.2.5 Buffer Exchange

Complete pH adjustment to the PBS pH (η = 1, outlet pH = 7.4) was achieved at flow rates ≤ 25 µL/min. At 500 µL/min, η dropped to 0.3 (pH ≈ 5.0), indicating insufficient residence time for citrate‐to‐phosphate buffer replacement. The pH‐adjustment process is slower than ethanol removal because of the buffering capacity of citrate and the lower diffusivity of the relevant ionic species relative to ethanol. Flow rate should therefore satisfy both ethanol‐removal (NTU > 4.6) and pH‐adjustment criteria. The latter typically sets the upper limit on practical flow rate.

#### 3.2.6 Design Guidelines

All parameter studies presented above reveal a clear hierarchy of design controls. Under the fixed‐flow‐rate conditions tested, channel height was the principal geometric design control in this system: The transverse‐diffusion time scales quadratically with height, so reducing the channel height is the most effective geometric means of increasing the channel‐side contribution to K_ov_. This does not imply that the channel‐side boundary layer is the largest of the three resistances in Equation (3). Rather, because the membrane resistance is fixed once the membrane is selected, channel height is the principal variable available for tuning. Accordingly, a 100 µm channel provides the most favorable geometry for achieving efficient ethanol removal and effective buffer exchange. In contrast, channel length and width serve mainly as practical design parameters. Increasing channel length enhances the available NTUs and can be readily implemented through serial concatenation, whereas variations in channel width have minimal impact on the rate‐limiting vertical transport. Membrane selection introduces an independent trade‐off between removal kinetics and particle recovery, as illustrated by the differences between PES and RC membranes.

Because pH adjustment and buffer exchange proceed more slowly than ethanol removal, they ultimately set the upper limit on the achievable flow rate. The central design principle is therefore to first minimize channel height to alleviate transverse diffusion limitations, and then adjust channel length, width, membrane type, and flow rate according to the desired throughput and recovery.

### 3.3 Comparison With Conventional Methods

**FIGURE 3:**
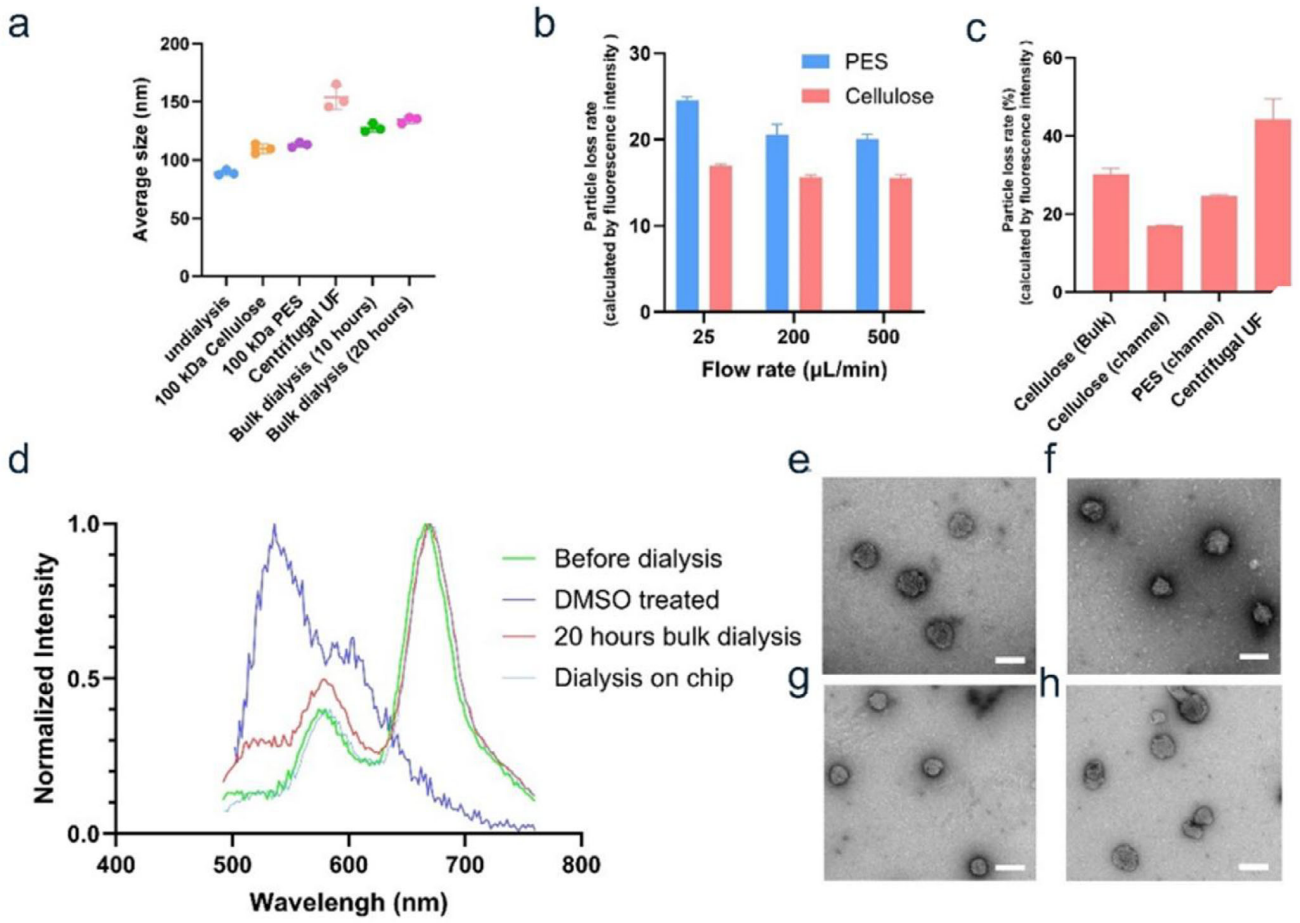
The purification performance and particle loss rate of different membrane materials and methods. (a) Average size of mRNA‐loaded LNPs before and after dialysis by different methods. (b) Particle loss rate (calculated from fluorescence intensity) at 25, 200, and 500 µL/min for PES (blue) and RC (red) membranes. (c) Comparison of particle loss rates for different methods. (d) Förster resonance energy transfer (FRET) assay monitoring LNP structural integrity after dialysis, including a DMSO‐treated sample as a disrupted‐particle control. (e–h) Representative TEM images of LNPs purified by (e) on‐chip dialysis using PES, (f) on‐chip dialysis using RC, (g) bulk dialysis, and (h) centrifugal ultrafiltration. Scale bars: 100 nm.

To investigate the purification efficiency of the microfluidic chip, two membrane materials (PES and RC) and different purification methods were investigated, including bulk dialysis, on‐chip dialysis, and centrifugal ultrafiltration. As shown in Figure 3a, unpurified LNPs showed smaller average particle sizes, while all purification methods used in this study tended to increase particle size.

The effect of dialysis on particle size has been reported inconsistently in the literature, with some studies showing an increase, while others reported a particle size reduction following dialysis [15]. This discrepancy may result from several factors. One likely contributor is the solvent‐exchange process itself. Removal of ethanol, together with neutralization of the medium from pH 4.0 to 7.4, may alter the protonation state of the ionizable lipid and the hydration environment of the lipid and PEG‐lipid components. These changes can promote structural reorganization during LNP maturation, consistent with previous reports showing that ionizable LNPs may undergo morphology changes as the pH is raised above the apparent pKa of the ionizable lipid [16]. This solvent‐exchange‐driven maturation may therefore contribute to the general size increase observed after purification. Method‐dependent effects, including prolonged exposure to ethanol and acidic conditions, membrane adsorption of smaller particles, particle fusion, aggregation, or shear‐induced damage, may then be superimposed on this baseline effect.

Slow dialysis can prolong exposure to ethanol and acidic pH, which may promote particle fusion or other maturation processes and thereby increase particle size. Conversely, preferential adsorption or loss of smaller nanoparticles on the dialysis membrane can also shift the measured average size upward. Rapid dialysis may better preserve the original size distribution, whereas mechanically aggressive conditions can induce nanoparticle deformation, aggregation, or partial disruption. Particle‐size changes should therefore be interpreted together with recovery and structural‐integrity measurements.

LNPs labelled with the lipophilic carbocyanine tracer DiI were then used to quantify particle recovery based on fluorescence intensity before and after dialysis. As shown in Figure 3b, at different flow rates (25, 200, and 500 µL/min), RC membranes showed lower LNP loss than PES membranes, consistent with the lower nonspecific adsorption of RC noted in Section 3.2.4. A broader comparison of particle recovery across methods is shown in Figure 3c; RC‐based on‐chip dialysis showed the lowest particle‐loss rate (around 17%), lower than those observed for centrifugal ultrafiltration and PES‐based on‐chip dialysis.

Further evaluation of LNP structural integrity was performed using a Förster resonance energy transfer (FRET) assay (Figure 3d) [17]. The DiO (donor)/DiI (acceptor) pair was incorporated into the LNP lipid phase. The sample before dialysis showed a characteristic FRET signal, indicating intact LNP structure. The DMSO‐treated sample showed a markedly quenched donor signal, consistent with complete structural disruption of LNPs [18]. Bulk dialysis (20 h overnight) resulted in reduced FRET efficiency, suggesting partial LNP disruption during purification. In contrast, the on‐chip dialysis sample maintained a FRET signal similar to that of the untreated control, indicating better preservation of LNP structure during dialysis.

TEM imaging confirmed that on‐chip dialysis with either PES or RC membrane produced uniform, spherical particles with smooth surfaces (Figure 3e,f), similar to bulk dialysis (Figure 3g). Centrifugal ultrafiltration samples showed occasional edge deformation and small aggregates (Figure 3h), consistent with mechanical stress during centrifugation. Although these morphological differences were qualitative, they support the FRET and recovery data indicating gentler processing by on‐chip dialysis.

Particle loss could be further reduced through optimization of both material interfaces and operating conditions. Membrane pre‐conditioning and surface passivation strategies may suppress nonspecific adsorption of LNPs, while the selection of low‐binding membrane chemistries can further minimize particle–membrane interactions. In addition, reducing dead volume and exposed internal surface areas would decrease opportunities for nanoparticle adsorption. Process optimization is also important, as operating within the minimum residence‐time window required for ethanol removal and pH adjustment can limit unnecessary membrane exposure while maintaining effective exchange performance. These strategies are compatible with the clamped‐membrane architecture because the membrane can be exchanged without re‐fabricating the chip.

**FIGURE 4:**
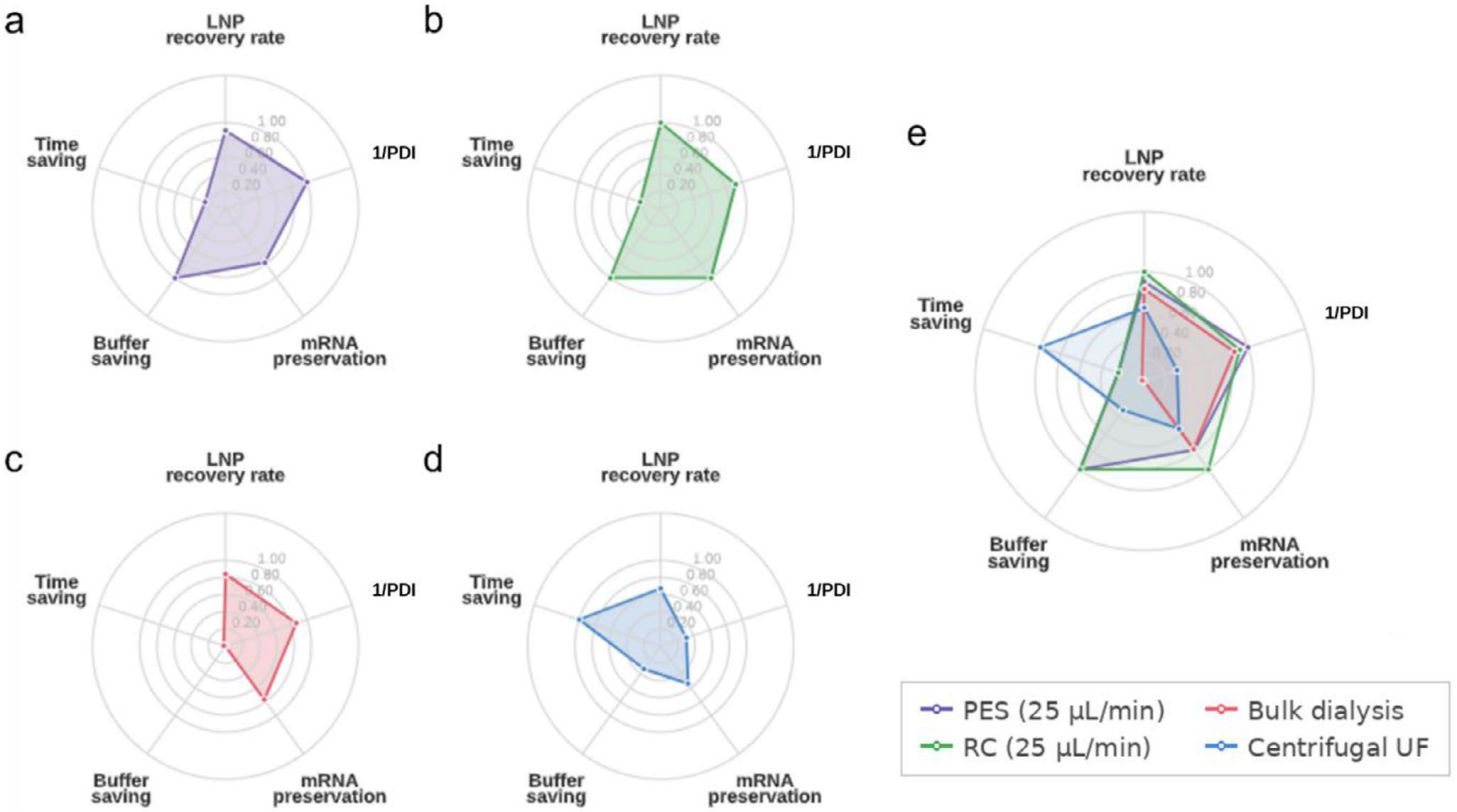
Radar‐plot comparison of four LNP purification methods across five normalized performance metrics. (a–d) Individual radar charts for (a) PES (25 µL/min), (b) RC (25 µL/min), (c) bulk dialysis, and (d) centrifugal ultrafiltration, showing reciprocal PDI (particle stability), LNP recovery rate (1 – loss), time saving (1/min), mRNA preservation rate, and buffer saving (1 – usage). (e) Overlay of all four methods for direct comparison. Raw metric values are given in Table S1; each metric is normalized to the maximum value observed across all methods.

To compare the trade‐offs among purification methods, we used radar plots based on five normalized performance metrics: reciprocal PDI (1/PDI, used here as an inverse indicator of dispersity), LNP recovery, time saving, mRNA preservation, and buffer saving (Figure 4). Raw metric values are provided in Table S1; for plotting, each metric was divided by the maximum value observed across all methods so that every axis ranges from 0 (worst) to 1 (best). PES (25 µL/min, Figure 4a) showed good particle stability, LNP recovery, mRNA preservation and buffer saving, but scored lower for time saving than centrifugal ultrafiltration. Compared with the PES membrane, the RC membrane showed slightly higher LNP recovery and mRNA preservation, but a lower particle‐stability score (Figure 4b). Conventional bulk dialysis (Figure 4c) showed low dispersity but had the longest processing time and highest buffer cost among all methods. Centrifugal ultrafiltration (Figure 4d) stood out for rapid processing, but the centrifugation‐based process resulted in lower particle quality. It also resulted in the lowest LNP recovery and mRNA retention among the evaluated purification approaches, as these samples exhibited greater particle loss and reduced mRNA preservation during centrifugal ultrafiltration, as reflected by the recovery and encapsulation data (Figure 3c and Table S1).

When all four methods are overlaid (Figure 4e), both on‐chip configurations showed the most balanced overall performance, combining high recovery and mRNA preservation with relatively low buffer usage and moderate processing times. Centrifugal ultrafiltration offered the greatest time saving but underperformed in recovery, particle quality, and mRNA preservation. Bulk dialysis preserved particle quality but required substantially longer processing time and higher buffer consumption. In summary, the on‐chip dialysis platform provides the most balanced performance among the methods tested. Consistent with Section 3.2.4, PES is recommended when rapid ethanol removal is the priority, while RC may be preferred when minimizing particle loss is the primary concern.

Compared with previously reported microfluidic buffer‐exchange platforms, the approach presented here offers a modular and readily adaptable solution. The fully 3D‐printed chip architecture eliminates the need for cleanroom lithography, while the mechanically clamped membrane design enables straightforward replacement of commercially available dialysis membranes without redesign or re‐bonding of the device. In addition, the NTU‐based design framework provides a transferable strategy for predicting and optimizing mass‐transfer performance across different channel geometries, rather than relying solely on device‐specific optimization. The capability of this platform is further demonstrated through the purification and buffer exchange of shear‐sensitive mRNA‐LNPs, with performance evaluated based on particle recovery, structural integrity, and retained transfection activity [19, 20]. In particular, mRNA‐LNPs undergo particle fusion and stepwise morphological rearrangement during buffer exchange as the pH is raised, and the buffer‐exchange method can influence the final particles [21], making a gentle, continuous, and controllable purification route especially valuable for this class of formulation.

### 3.4 In Vitro Validation

**FIGURE 5:**
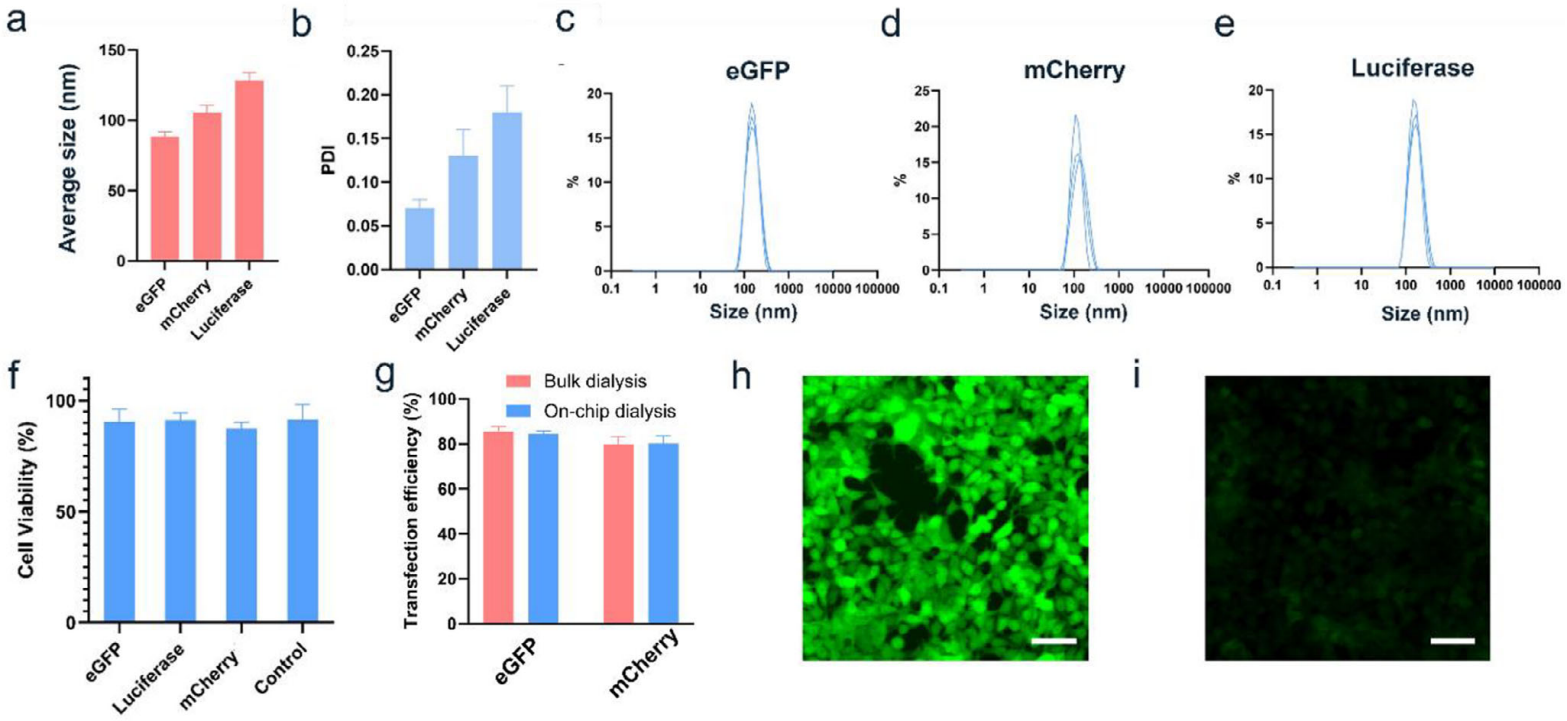
Characterization and in vitro performance of different mRNA‐loaded LNP formulations. (a) Average hydrodynamic diameter of LNPs encapsulating eGFP, mCherry, or luciferase mRNA. (b) PDI for each formulation. (c–e) Representative size‐distribution profiles of LNPs loaded with (c) eGFP, (d) mCherry, and (e) luciferase mRNA. (f) Cell viability of HEK293 cells after 24 h treatment with LNPs (eGFP, luciferase, or mCherry) or untreated control. (g) Transfection efficiency of eGFP‐ and mCherry‐loaded LNPs purified by bulk dialysis or on‐chip dialysis, expressed as the percentage of fluorescent‐protein‐positive cells after 24 h incubation, as determined by flow cytometry. Data are mean ± SD (*n* = 3). Representative fluorescence microscopy images of transfected cells (eGFP) with mRNA LNPs (h) and untreated cells without adding mRNA LNPs as the negative control (i). Scale bars: 50 µm.

To evaluate biological performance, three types of mRNA were encapsulated in LNPs and purified using the optimized on‐chip protocol. As shown in Figure 5a,b, on‐chip purification produced LNPs with consistent sizes and low PDI values. Particle size increased with mRNA length, while PDI values remained below 0.25 for all formulations. Figure 5c–e further confirmed these observations, showing sharp, unimodal size‐distribution peaks without secondary populations or visible aggregates.

To further assess the biological performance of the purified mRNA‐LNPs, we evaluated cytotoxicity and transfection in HEK293 cells. The potential cytotoxicity was tested after co‐incubation of cells with the mRNA‐loaded LNPs. WST measurements at 490 nm showed >90% viability for all formulations, with no detectable difference from untreated controls (Figure 5f).

Flow cytometry indicated transfection efficiencies of 80%–86% for both eGFP‐ and mCherry‐loaded LNPs (Figure 5g). On‐chip purification produced values comparable to those obtained after bulk dialysis. Luciferase activity was also largely retained after on‐chip purification: the on‐chip and bulk‐dialysis groups yielded 141,369 ± 818 and 146,366 ± 335 relative light units (RLU), respectively, with the on‐chip value being 3.4% lower than that of the bulk‐dialysis group. Fluorescence microscopy images also showed strong and uniform fluorescent protein expression in transfected cells, with minimal background signal in untreated controls (Figure 5h,i and Figure S1). Taken together, these biological results support the compatibility of the on‐chip purification process with functional mRNA‐LNP delivery. The microfluidic dialysis chip operates under gentle laminar‐flow conditions, which reduces shear exposure while enabling efficient ethanol removal and effective buffer exchange under continuous‐flow operation. This processing environment helps maintain the narrowly distributed particle population (PDI < 0.25) and the LNP structural integrity demonstrated in Section 3.3. These attributes, together with the low cytotoxicity observed in HEK293 cells, are important for maintaining efficient cellular uptake and mRNA expression. Together, the flow‐cytometric transfection efficiencies for eGFP and mCherry and the largely retained luciferase activity indicate that the on‐chip purification process preserved the functional performance of the LNP formulations.

## 4 Conclusion

A microfluidic dialysis chip was developed for LNP purification, and a mass transfer framework was established to guide its design. The chip employs a three‐layer sandwich structure with serpentine counter‐current flow, providing efficient ethanol removal and effective buffer exchange under gentle laminar flow conditions. Under the fixed‐flow‐rate conditions tested, systematic parameter optimization identified channel height as the principal geometric variable governing mass‐transfer performance. Dimensionless analysis indicated that efficient operation was associated with a transverse‐diffusion time substantially shorter than the residence time; at H = 100 µm and the operating flow rate, τ_res_/τ_d_ was approximately 11. Channel width had negligible effect, while channel length directly scaled NTU. Buffer exchange and pH adjustment, rather than ethanol removal alone, set the upper limit on practical flow rates.

Under optimized conditions, the chip achieved >99% ethanol removal and up to 83% particle recovery while processing 2 mL of samples within 80 min. This recovery exceeded that of centrifugal ultrafiltration (56%) at a comparable processing speed and was achieved in substantially less time than bulk dialysis. Purified mRNA‐LNPs retained structural integrity; eGFP‐ and mCherry‐loaded LNPs achieved transfection efficiencies of 80%–86% in vitro, while luciferase‐loaded LNPs showed largely retained reporter activity compared with bulk dialysis. The modular architecture theoretically supports throughput adjustment through parallelization or serial concatenation. Overall, the NTU‐based framework and design guidelines established here provide a rational foundation for adapting microfluidic dialysis to a range of LNP purification requirements.

5

**Nomenclature**
A: Membrane contact area, m^2^
C: Ethanol concentration in sample channel, mol/m^3^
C_in_
: Inlet ethanol concentration, mol/m^3^
C_out_
: Outlet ethanol concentration, mol/m^3^
C_b_
: Ethanol concentration in dialysate (buffer) channel, mol/m^3^
D: Diffusion coefficient of ethanol in water, m^2^/s
d_h_
: Hydraulic diameter, m
H: Channel height, m
K_ov_
: Overall mass transfer coefficient, m/s
k_s_
: Sample‐side mass transfer coefficient, m/s
k_m_
: Membrane mass transfer coefficient, m/s
k_b_
: Buffer‐side mass transfer coefficient, m/s
L: Channel length, m
NTU: Number of transfer units, dimensionless
Pe_t_
: Transverse Péclet number, dimensionless
Q: Volumetric flow rate, m^3^/s
Sh: Sherwood number, dimensionless
u: Average flow velocity, m/s
W: Channel width, m
τ_d_
: Characteristic diffusion time, s
τ_res_
: Residence time, s
η: Buffer exchange efficiency, dimensionless

## Author Contributions

**Da Zou**: conceptualization, methodology, software, investigation, data curation, formal analysis, validation, visualization, writing – original draft. **Zhenwei Lan**: methodology, investigation, writing – review & editing. **Jingwen Liu**: investigation, writing – review. **Sha Liu**: investigation, writing – review & editing. **Song Jin**: investigation, writing – review & editing. **Yang Li**: investigation, writing – review & editing. **Phillip Elliott**: conceptualization, writing – review & editing. **Jan Bekker**: conceptualization, writing – review & editing. **Chun‐Xia Zhao**: conceptualization, methodology, supervision, resources, project administration, writing – review & editing.

## Conflicts of Interest

The authors declare no conflicts of interest.

## Supporting information




**Supporting File**: smtd70976‐sup‐0001‐SuppMat.docx.

## Data Availability

The data that support the findings of this study are available from the corresponding author upon reasonable request.
