# Supplementary material for: A Microfluidic Dialysis Chip for Continuous Purification of Lipid Nanoparticles
Source: Small Methods. 2026 Aug 18;10(18):e70976. doi: 10.1002/smtd.70976 (PMC13599676; doi:10.1002/smtd.70976)
Supplement: Supplementary file 1 [file SMTD-10-e70976-s001.docx]

**Supporting Information**

**Table S1. Metric values used for the radar plots, prior to column-wise normalisation**

| Method | 1/PDI | LNP recovery rate (1 - loss rate) | Time saving (1/min) | Buffer saving (relative metric) | mRNA preservation rate |
| --- | --- | --- | --- | --- | --- |
| PES (25μL/min) | 8.33 | 0.755 | 0.01 | 1 | 0.694 |
| RC (25μL/min) | 7.66 | 0.83 | 0.01 | 1 | 0.89 |
| Bulk dialysis | 7.23 | 0.698 | 0.00083 | 0.001 | 0.685 |
| Centrifugal UF | 2.63 | 0.557 | 0.04 | 0.33 | 0.482 |

Note: The tabulated values are the per-metric quantities used for the radar plots in Fig. 4, before column-wise normalisation; for the plots, each metric was divided by the maximum value in its column so that every axis ranges from 0 to 1, with higher values indicating better performance. PDI and processing time are expressed as reciprocals (1/PDI and 1/min) so that larger values correspond to better performance.

**Fig. S1.** Representative fluorescence microscopy images of transfected cells, (a): mCherry-LNP treated and (b): blank. Scale bar: 50 μm.

**Supplementary Methods. Convection–diffusion modelling of the dialysis chip**

We used a convection–diffusion description of the chip to rationalise three design choices: the counter-current configuration, the role of channel height at fixed volumetric flow rate, and the dialysate-to-sample flow ratio. The models are mechanistic and parametric; the transport coefficients were set to representative values rather than fitted to the measurements, so the results are intended to explain the observed trends rather than to reproduce individual data points.

**Two-stream contactor (Fig. S2 and S4).** The sample and wash-buffer streams are treated as plug flows separated by the membrane, with a lumped overall mass-transfer coefficient K_ov_. Axial ethanol conservation in the sample stream gives Q_s_·dC_s_/dx = −K_ov_·w·(C_s_ − C_d_), and the dialysate stream obeys Q_d_·dC_d_/dx = ±K_ov_·w·(C_s_ − C_d_), with the plus sign for co-current and the minus sign for counter-current operation, where w is the channel width, x the axial coordinate, and C_d_ the local dialysate concentration. We set K_ov_·w·L/Q_s_ = NTU = 4.6, the value corresponding to 99% removal in the limit of an infinitely dilute dialysate. The counter-current case was solved as a boundary-value problem (C_s_ = C_in_ at the sample inlet, C_d_ = 0 at the dialysate inlet) and the co-current case as an initial-value problem. Fig. S2 compares the two configurations at a dialysate-to-sample flow ratio of 5:1, and Fig. S4 varies this ratio from 1:1 to 10:1.

**Two-dimensional channel (Fig. S3).** To resolve transport across the channel height, the sample channel was modelled in two dimensions (axial x, transverse y) with a fully developed parabolic velocity profile u(y) = 6U(y/H)(1 − y/H), where U = Q/(wH) is the mean velocity and H the channel height. Neglecting axial diffusion (Péclet number ≫ 1), the steady scalar-transport equation is u(y)·∂C/∂x = D·∂²C/∂y², with D = 1.2 × 10⁻⁹ m² s⁻¹ for ethanol in water at 25 °C. The membrane (y = 0) was imposed as a Robin flux boundary, −D·∂C/∂y = k_m_(C − C_d_) with C_d_ = 0 and k_m_ = 5 × 10⁻⁶ m s⁻¹; the opposite wall (y = H) was treated as no-flux; and C = C_in_ was imposed at the inlet. The equation was integrated along x with an implicit (Thomas-algorithm) treatment of the transverse diffusion term. Fig. S3 compares H = 100 and 500 μm at the operating flow rate (25 μL min⁻¹), showing both the concentration field and the flow-weighted (cup-mixing) outlet concentration along the channel.

**Geometry, parameters and implementation.** The channel length (L = 38.4 cm), width (w = 1 mm) and membrane area were taken from the fabricated device. An OpenFOAM (scalarTransportFoam) implementation of the two-dimensional case, using the same Robin membrane boundary condition, is provided with the associated data and reproduces the results of Fig. S3.

**Limitations.** The models assume steady state, dilute and isothermal transport, constant coefficients, and no membrane fouling or particle effects; the two-stream model further assumes a well-mixed cross-section. They therefore capture the transport mechanisms underlying the design guidelines but are not intended as quantitative predictions of ethanol removal for a specific formulation.

**Fig. S2.** Counter-current versus co-current operation of the dialysis chip, from the two-stream contactor model (dialysate-to-sample flow ratio Q_d_:Q_s_ = 5:1, NTU = 4.6). (a) Sample and dialysate ethanol concentration profiles along the channel. (b) Local trans-membrane driving force. Counter-current operation maintains the driving force along the whole channel and reaches an outlet concentration of about 2% of the inlet, whereas the co-current driving force collapses toward the outlet, leaving about 17%.

**Fig. S3.** Two-dimensional convection–diffusion field in the sample channel at matched volumetric flow rate. (a, b) Normalised ethanol concentration C/C_in_ for channel heights of 100 and 500 μm; the membrane is the lower boundary of each panel. (c) Flow-weighted (cup-mixing) outlet concentration along the channel. The 100 μm channel equilibrates across its height, whereas the 500 μm channel retains a high-concentration core near the free wall, giving poorer removal at the same flow rate.

**Fig. S4.** Effect of the dialysate-to-sample flow ratio on the counter-current chip. (a) Dialysate-side ethanol build-up along the channel for ratios of 1:1, 5:1 and 10:1. (b) Peak dialysate concentration as a function of the flow ratio. A 1:1 ratio approaches saturation (about 82% of the inlet concentration at the concentrated end), whereas 5:1 keeps the dialysate below about 20% and 10:1 below about 10%, justifying the 5:1 ratio used in this work.
